# Secondary Intracranial Hypertension Mimicking Meningitis in an Immunosuppressed Patient With Systemic Lupus Erythematosus

**DOI:** 10.7759/cureus.105996

**Published:** 2026-03-27

**Authors:** Amara Liaqat, Cristina Vladulescu

**Affiliations:** 1 General Medicine, Aberdeen Royal Infirmary, NHS Grampian, Aberdeen, GBR

**Keywords:** cerebrospinal fluid opening pressure, intracranial hypertension, meningitis mimic, papilloedema, systemic lupus erythematosus

## Abstract

Intracranial hypertension can mimic acute meningitis, especially in immunosuppressed patients presenting with fever and meningism, often prompting urgent empirical treatment. Distinguishing infectious from non-infectious causes is crucial to avoid unnecessary antimicrobials and delayed diagnosis. We report a 20-year-old woman with systemic lupus erythematosus on rituximab and a recent corticosteroid taper who presented with fever, severe headache, photophobia, and neck stiffness. She was started on empirical antibiotics for suspected meningitis. Lumbar puncture showed a markedly raised opening pressure (43 cm H2O) with otherwise normal cerebrospinal fluid (CSF). Neuroimaging excluded structural lesions and cerebral venous sinus thrombosis, while ophthalmologic examination confirmed papilloedema. Her symptoms improved after a therapeutic lumbar puncture and acetazolamide. This case highlights the value of measuring CSF opening pressure and reconsidering the diagnosis in immunosuppressed patients with meningitic features.

## Introduction

Systemic lupus erythematosus (SLE) is a chronic multisystem autoimmune disease with diverse neurological manifestations. Neuropsychiatric involvement may range from headache and cognitive dysfunction to seizures, cerebrovascular events, aseptic meningitis, and inflammatory syndromes affecting the central nervous system. More recent reviews emphasize that neurological presentations in SLE are often heterogeneous, relapsing, and diagnostically challenging, especially when immunosuppressive treatment alters the usual inflammatory response or obscures classical clinical patterns [1,2]. In this setting, fever and meningeal irritation may lead clinicians toward infection first, which is understandable and often appropriate in the acute phase.

Raised intracranial pressure without mass lesion, hydrocephalus, or abnormal cerebrospinal fluid (CSF) composition is an uncommon but recognized complication in SLE. The overlap between intracranial hypertension and meningitis is clinically important because both may present with severe headache, photophobia, nausea, neck stiffness, and visual symptoms. In immunosuppressed patients, the threshold for empirical antibiotics must remain low because delayed treatment of bacterial meningitis carries substantial morbidity and mortality. However, immunosuppression can also create diagnostic bias: clinicians may anchor on infection despite normal CSF indices or rapidly improving symptoms after CSF drainage [2-4]. This case illustrates how careful interpretation of lumbar puncture findings, ophthalmological examination, and venous imaging can redirect diagnosis toward secondary intracranial hypertension and avert preventable visual complications.

## Case presentation

A 20-year-old woman with established SLE and biopsy-confirmed class II lupus nephritis presented with a 48-hour history of progressively worsening severe headache, photophobia, neck stiffness, fever (38.5°C), and visual blurring. She was receiving rituximab therapy and had recently undergone a corticosteroid taper. This was her third admission with a similar clinical presentation.

On admission, she was alert and oriented with a Glasgow Coma Scale score of 15. There were no focal neurological deficits. Because the combination of fever, headache, photophobia, and neck stiffness raised immediate concern for acute meningitis, empirical intravenous ceftriaxone and dexamethasone were administered after initial assessment.

**Figure 1:**
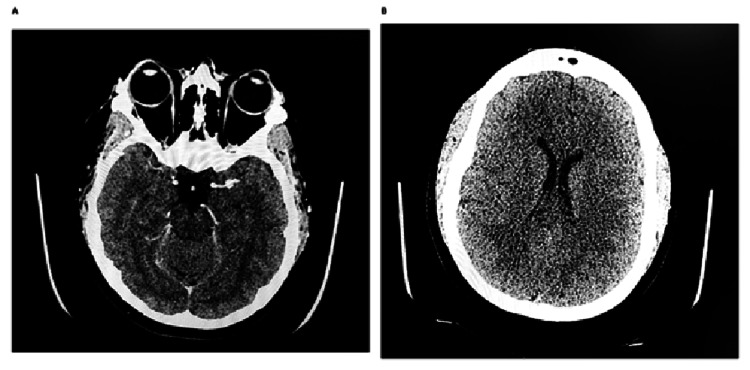
CT venography and CT of the head Neuroimaging during admission. (A) CT venography demonstrating no evidence of cerebral venous sinus thrombosis. (B) Axial CT of the head demonstrating no acute intracranial abnormality, mass effect, or extra-axial collection. CT: computed tomography

Computed tomography (CT) venography (Figure 1A) was then performed and did not demonstrate cerebral venous sinus thrombosis. CT of the brain (Figure 1B) showed no acute structural abnormality. Magnetic resonance imaging (MRI) venogram was normal. Lumbar puncture revealed a markedly elevated opening pressure of 43 cm H2O, while CSF cell count, protein, glucose, and microbiological studies were all normal. CSF was clear and colourless. CSF microscopy showed red blood cells at 3/µL and white blood cells at 26/µL. Gram stain showed no organisms, and CSF culture showed no growth. Antigen testing was negative for group B *Streptococcus*, *Haemophilus influenzae* group b, *Streptococcus pneumoniae*, *Neisseria meningitidis* serogroups A/C/Y/W135, and meningococcal B/*Escherichia coli* K1. Eye swab microscopy showed no pus cells or organisms, and culture demonstrated no pathogens isolated. The closing pressure was 20 cm H2O after the removal of 30 mL of CSF. The headache improved significantly after therapeutic CSF drainage. Subsequent ophthalmologic review demonstrated visual acuity of 6/6 in both eyes, intraocular pressure of 15 mmHg in the right eye and 11 mmHg in the left eye, and mild bilateral disc swelling, greater on the right, consistent with Frisén grade 1 papilloedema and anterior uveitis. Visual fields were satisfactory. Acetazolamide was commenced at 250 mg twice daily, which was increased to 500 mg twice daily after one week. At follow-up, the dose had been escalated to 500 mg twice daily and was continued with subsequent clinical stabilization.

The clinical timeline therefore supported a non-infectious pressure-mediated process: symptom onset over 48 hours, empirical treatment on admission because meningitis could not safely be excluded, and diagnostic lumbar puncture showing isolated intracranial pressure elevation with otherwise normal CSF. Taken together, these findings favoured secondary intracranial hypertension rather than bacterial meningitis. Also, the headache had significantly improved, with only mild retro-orbital pressure and no further photophobia. At the vasculitis clinic follow-up, she remained stable; acetazolamide had been increased to 500 mg twice daily and was continued, with ongoing specialist follow-up arranged (Table 1). 

**Table 1:** Key diagnostic findings CT: computed tomography; CSF: cerebrospinal fluid

Investigation	Result
CT of the brain	Normal
CT venography	No cerebral venous sinus thrombosis
CSF opening pressure	43 cm H2O
CSF cell count	Normal
CSF protein/glucose	Normal
Microbiology	Negative

## Discussion

This case illustrates the diagnostic complexity of acute neurological presentations in immunosuppressed patients with SLE. The initial decision to treat for meningitis was appropriate because bacterial meningitis is time-sensitive and potentially fatal and early empirical antimicrobial therapy is standard when suspicion is high. However, several features subsequently argued against meningitis, including normal CSF cell counts, normal biochemical parameters, absence of microbiological evidence of infection, and rapid symptomatic improvement after CSF drainage. These red flags against meningitis increased the educational value of the case by showing how lumbar puncture findings can redirect the diagnosis toward a pressure-mediated process rather than infection [4].

The differential diagnosis in this scenario included bacterial meningitis, viral or aseptic meningitis related to SLE, cerebral venous sinus thrombosis, lupus-related central nervous system inflammation, and intracranial hypertension. Cerebral venous sinus thrombosis was especially important to exclude because it can present with headache, papilloedema, and elevated intracranial pressure, particularly in inflammatory or prothrombotic states. The absence of abnormal CSF indices and the negative venographic study supported secondary intracranial hypertension as the most coherent final diagnosis [4-7].

Intracranial hypertension has been reported in association with SLE, although it remains uncommon. Proposed mechanisms include immune-mediated endothelial dysfunction, altered cerebral venous outflow, inflammatory effects on CSF absorption, and medication-related factors such as corticosteroid exposure or withdrawal [2,3,8]. The patient's previous similar episodes may suggest a recurrent intracranial hypertension phenotype rather than an isolated event, although definitive classification would require longitudinal follow-up and the exclusion of other secondary causes [5-7].

From an educational perspective, this case demonstrates why CSF opening pressure should be documented whenever a lumbar puncture is performed for suspected meningitis, provided it is safe to do so. In addition, exact visual acuity and formal papilloedema grading would have further strengthened reproducibility for future readers, especially when intracranial hypertension is part of the differential diagnosis [4-7].

A practical limitation is that MRI of the brain with venography is generally more sensitive than CT-based imaging for some alternative intracranial pathologies, although CT venography remains an accepted and useful initial investigation when venous sinus thrombosis must be excluded urgently [4,5].

## Conclusions

This case demonstrates that secondary intracranial hypertension in SLE can closely mimic meningitis at presentation. In this patient, raised CSF opening pressure, negative microbiological studies, absence of cerebral venous sinus thrombosis on imaging, and improvement following therapeutic lumbar puncture and acetazolamide supported a diagnosis of secondary intracranial hypertension.
